# Supplementary material for: Dielectric catastrophe at the Wigner-Mott transition in a moiré superlattice
Source: Nat Commun. 2022 Jul 25;13:4271. doi: 10.1038/s41467-022-32037-1 (PMC9314335; doi:10.1038/s41467-022-32037-1)
Supplement: Supplementary file 1 — Supplementary Information [file 41467_2022_32037_MOESM1_ESM.pdf]

Supplementary Information for
"Dielectric catastrophe at the Wigner-Mott transition in a moiré superlattice"

Yanhao Tang*, Jie Gu, Song Liu, Kenji Watanabe, Takashi Taniguchi, James C. Hone, Kin Fai Mak*, and Jie Shan*

These authors contributed equally: Yanhao Tang, Jie Gu
*Email: yanhaotc@zju.edu.cn; kinfai.mak@cornell.edu; jie.shan@cornell.edu

Supplementary figures

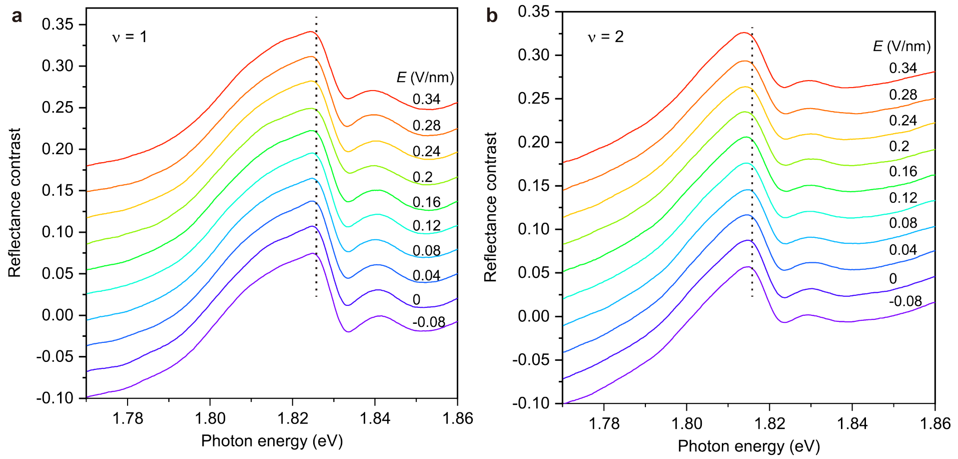

Supplementary Figure 1 | Electric-field dependence at $\nu = 1$ and $\nu = 2$. The reflectance contrast spectrum near the sensor 2s exciton under representative electric fields at $\nu = 1$ (**a**) and $\nu = 2$ (**b**). The spectra are vertically displaced by a constant 0.03 for clarity. The vertical dotted lines trace the 2s exciton peak. Negligible electric-field dependence is observed.

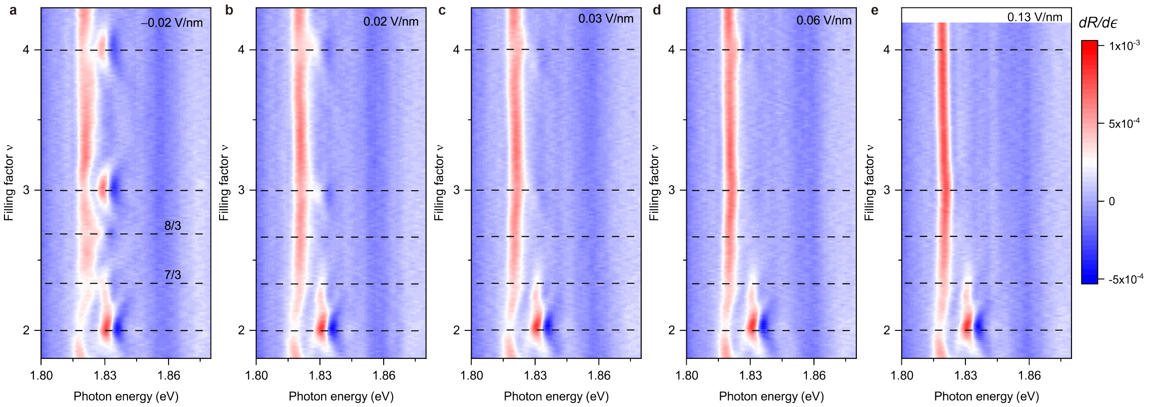

Supplementary Figure 2 | Electric-field tuned MITs in device 2. a-e, The energy-derivative of the reflectance contrast spectrum ($dR/d\epsilon$) of the sensor 2s exciton as a function of electron filling factor (ν = 2 - 4) at electric field E ranging from -0.02 V/nm to 0.13 V/nm. The incompressible states at E = -0.02 V/nm are labeled by the dashed black lines. They gradually dissolve as electric field increases. The $\nu = 4$ and $\nu = 3$ insulating states disappear at a similar electric field in this device.

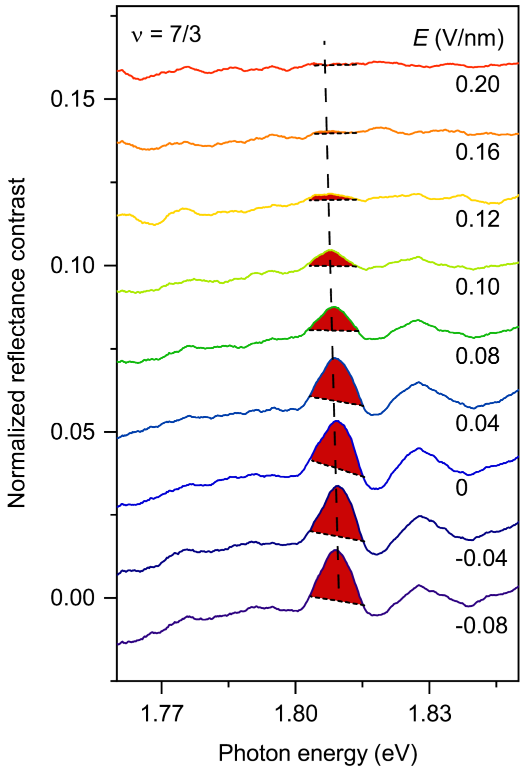

Supplementary Figure 3 | Normalized reflectance contrast spectra of the sensor 2s exciton at representative electric fields at $\nu = 7/3$. The reflectance contrast spectra are normalized to that at 0.24 V/nm, under which no incompressible states can be identified. The 2s exciton oscillator strength (shaded area) decreases to zero at large electric fields. The spectra are vertically displaced by a constant 0.02. The dashed line traces the 2s exciton peak.

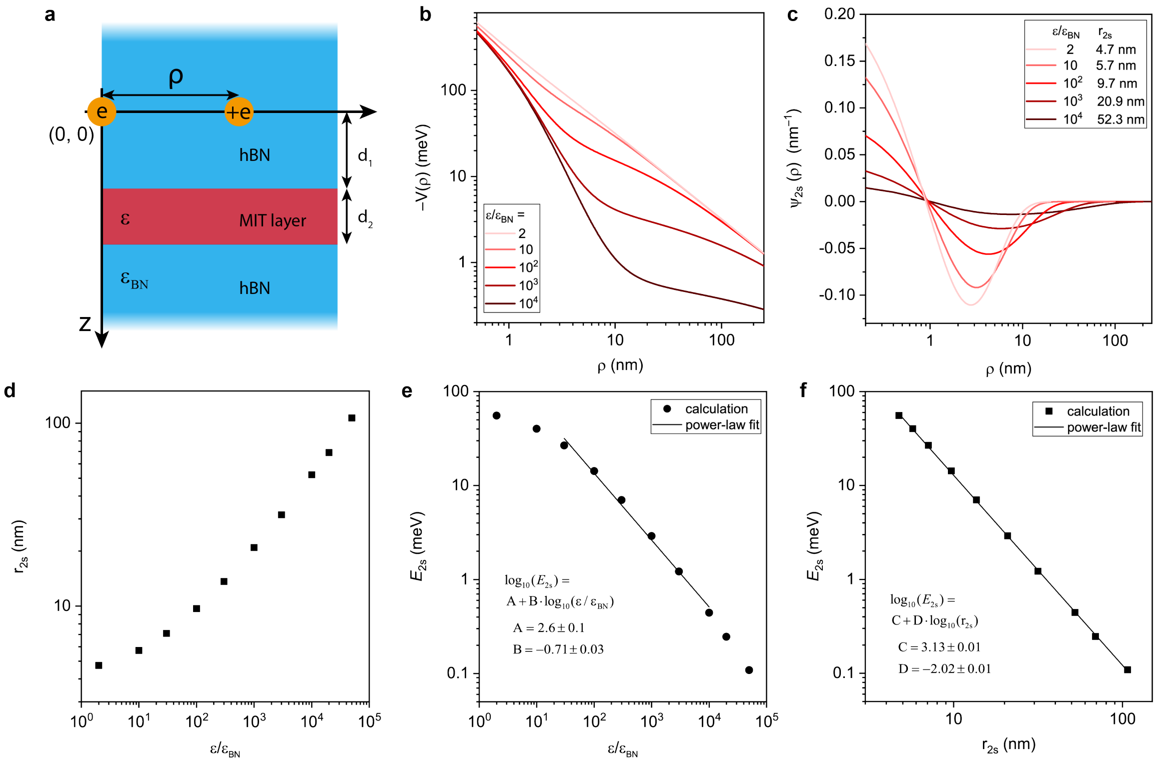

Supplementary Figure 4 | Modeling the 2s exciton Rydberg state. a, Schematic structure of the device geometry. The graphite gates are assumed to be far away from the excitons and are not shown. An electron-hole pair with separation ρ in the sensor layer is separated from the moiré sample (with thickness d_2) by a distance d_1. The sample and the sensor are both encapsulated in hBN (blue). d_1 and d_2 are set at 0.9 nm and 0.6 nm, respectively, to represent the realistic device geometry. **b**, **c**, Dependence of the screened electron-hole Coulomb potential $-V(\rho)$ (**b**) and the 2s exciton wavefunction $\Psi_{ns}(\rho)$ (**c**) on the electron-hole separation ρ for representative ratios of the dielectric constants $\frac{\varepsilon}{\varepsilon_{BN}}$. **d, e**, Dependence of the 2s Bohr radius (**d**) and the 2s binding energy on the ratio $\frac{\varepsilon}{\varepsilon_{BN}}$. The black line is a power-law fit to the result for $\frac{\varepsilon}{\varepsilon_{BN}}$ between 10^2 and 10^4. **f**, Dependence of the 2s binding energy on the 2s Bohr radius. The black line is a power-law fit to the result.

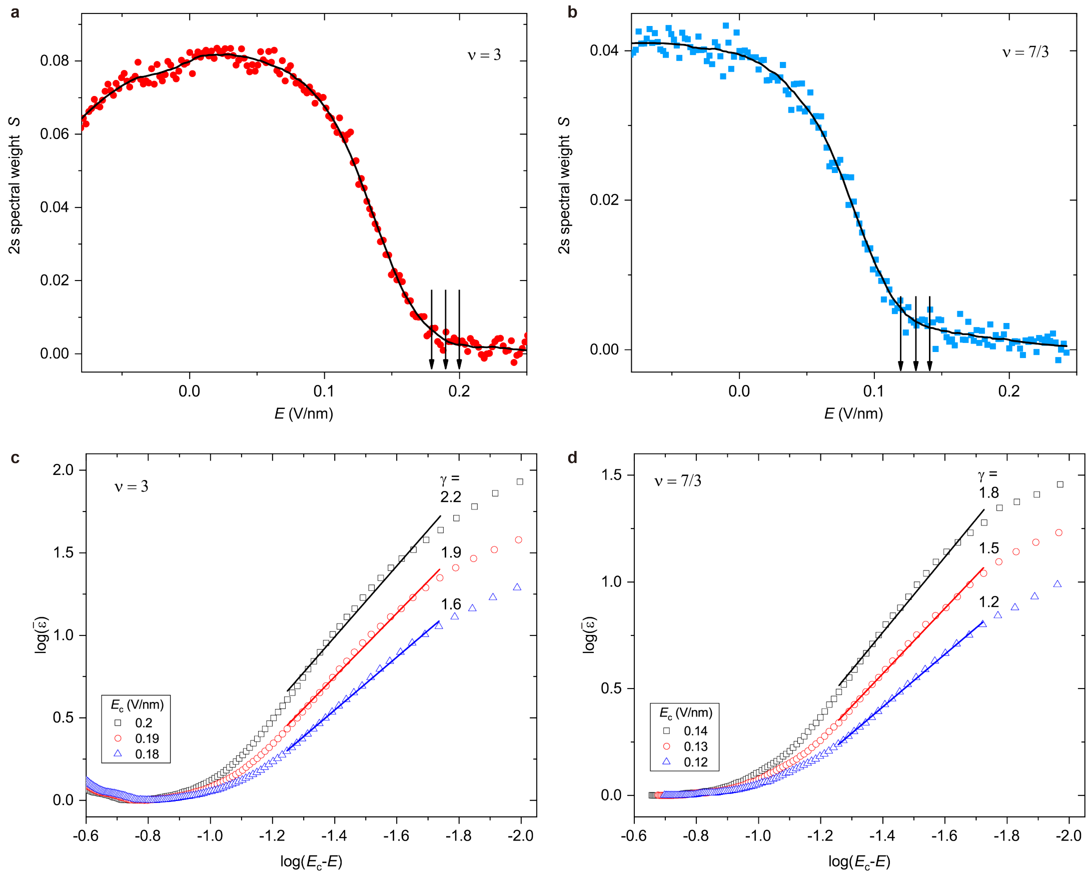

Supplementary Figure 5 | Critical exponents for the MITs. a, **b**, The 2s exciton spectral weight as a function of the electric field at $\nu = 3$ (**a**) and $7/3$ (**b**). The solid lines are the smoothed data using the Savitzky-Golay algorithm with a window of 80 mV/nm. The arrows denote the chosen critical electric fields E_c of the MITs; the value of E_c cannot be determined accurately in our experiment. **c**, **d**, Dependence of the normalized dielectric constant $\bar{\varepsilon}$ on the reduced electric field $E - E_c$ in log-log scale for $\nu = 3$ (**c**) and $7/3$ (**d**). The solid lines are linear fits to the data near E_c over a limited range of $E - E_c$ in order to estimate the value of the critical exponent γ, which varies from 1.6 to 2.2 for $\nu = 3$ and from 1.2 to 1.8 for $\nu = 7/3$. Different colors correspond to different chosen values of E_c.

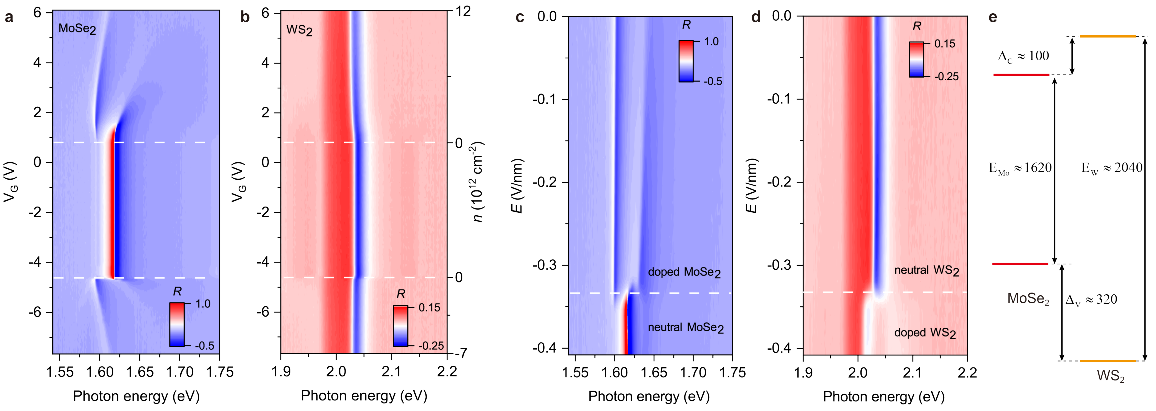

Supplementary Figure 6 | Band alignment in large-twist-angle $MoSe_2/WS_2$. a, **b**, The doping density dependent reflectance contrast spectrum of the $MoSe_2$ (**a**) and WS_2 (**b**) intralayer excitons under zero electric field. The gate voltage V_G here only changes the doping density (n). The white dashed lines indicate the onset of electron and hole doping. Charged excitons are only observed in the $MoSe_2$ layer, consistent with a type-I band alignment in $MoSe_2/WS_2$ heterobilayers. **c**,**d**, The electric-field dependent reflectance contrast spectrum of the $MoSe_2$ (**c**) and WS_2 (**d**) intralayer excitons at a constant electron doping density 3.7×10^{12} cm^{-2}. The white dashed lines, where the transition from charged exciton to neutral exciton occurs, correspond to the onset of charge transfer between $MoSe_2$ and WS_2. The corresponding electric field is used to determine the band offsets. **e**, The determined band alignment in $MoSe_2/WS_2$ heterobilayers (Methods). Energies are denoted in meV. E_{Mo} and E_W are the optical gaps of $MoSe_2$ and WS_2, respectively. Δ_C and Δ_V are the conduction and valence band offsets, respectively.

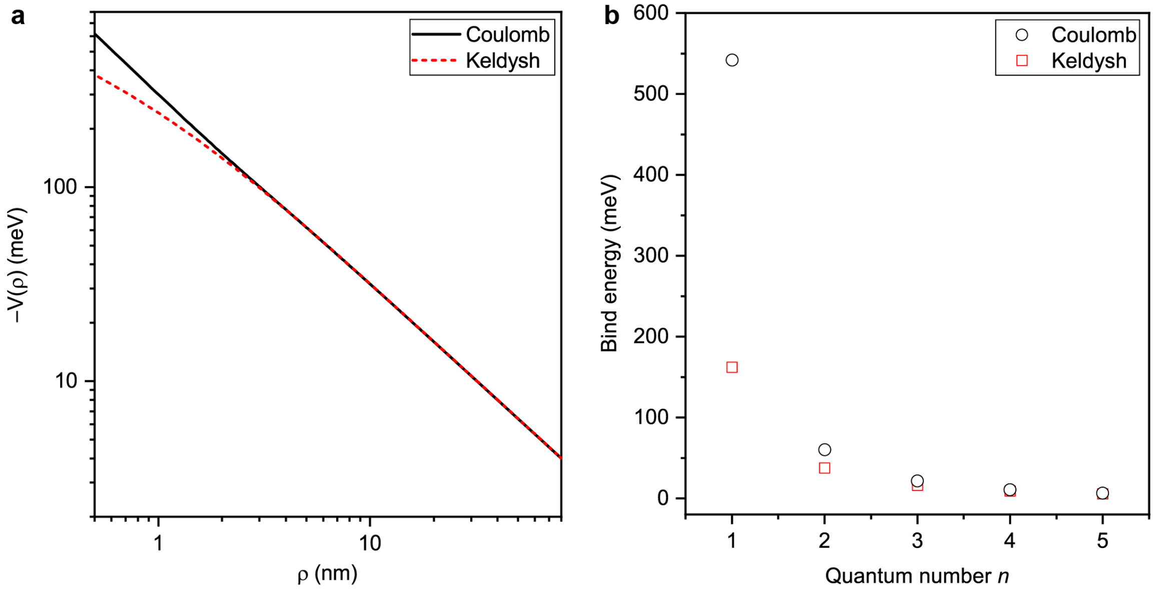

Supplementary Figure 7 | Comparison between the Coulomb and Rytova-Keldysh potential. **a**, The Coulomb and Rytova-Keldysh potential as a function of the electron-hole distance in a 2D plane (dielectric constant = 4.5). Substantial difference is observed only for distances smaller than the 1s exciton Bohr radius ~ 2 nm. **b**, The exciton binding energy as a function of the principle quantum number for the Coulomb and the Rytova-Keldysh potentials. Small difference is observed for $n \geq 2$.
